# Mental Capacity Law, Autonomy, and best Interests: An Argument for Conceptual and Practical Clarity in the Court of Protection

**DOI:** 10.1093/medlaw/fww034

**Published:** 2016-12-22

**Authors:** John Coggon

**Affiliations:** University of Bristol Law School, Bristol, UK

**Keywords:** Mental capacity, best interests, autonomy, medical decision-making, Court of Protection

## Abstract

This article examines medical decision-making, arguing that the law, properly understood, requires where possible that equal weight be given to the wishes, feelings, beliefs, and values of patients who have, and patients who are deemed to lack, decision-making capacity. It responds critically to dominant lines of reasoning that are advanced and applied in the Court of Protection, and suggests that for patient-centred practice to be achieved, we do not need to revise the law, but do need to ensure robust interpretation and application of the law. The argument is based on conceptual analysis of the law’s framing of patients and medical decisions, and legal analysis of evolving and contemporary norms governing the best interests standard.

## 1. Introduction

This article aims to correct some problematic jurisprudential positions that have developed in the Court of Protection. It responds in particular to two influential judgments: Lewison J’s decision in *Re P*,^1^ which *inter alia* purports to explain the ‘general philosophy’ of the Mental Capacity Act 2005 (MCA)^2^; and Baker J’s ruling in *Re M*, a case that reportedly addressed for the first time the question of whether the best interests of a patient in a minimally conscious state (MCS) were not served by continued provision of life-sustaining food and hydration.^3^ Baker J’s reasoning, with which this article directly engages, relies on that of Lewison J. However, it bears noting that a tension exists between their analyses: Lewison J suggests that the MCA *radically reformed* mental capacity law, while Baker J recognises the MCA as statutory codification within an *evolving* area of legal doctrine.^4^ Both judges, though, are united in denying full weight to incapacitated patients’ wishes, feelings, beliefs, and values under section 4(6) MCA.^5^ In essence, they hold that the values of patients who lack capacity cannot be determinative in the way that they are for patients with capacity because that would mean replacing the best interests standard with a substituted judgment test. This article challenges their claims.

If successful, the article supports what we may hope is a shift to more appropriate judicial approaches to section 4(6).^6^ Furthermore, it substantiates and explicates the underpinnings to relevant rulings in the Court of Appeal in *Burke v GMC*^7^ and the Supreme Court in *Aintree v James*,^8^ the latter of which, as I will argue, seems incompatible with *P* and *M*. My central claim is that mental capacity law has been devised with a commitment to achieving patient-centred care; care that honours where possible the patient’s own, reflectively endorsed values, whether or not she has decision-making capacity. This position is consistent with dominant themes in medical ethics and law,^9^ and prevailing national and international discourses.^10^ I will demonstrate how, all things equal, if a patient’s reflectively endorsed view on her interests is known, legally this should hold equal weight regardless of whether she has capacity or not.

To reach that conclusion, the article aims first for conceptual clarity. Section II explains the falsity of a binary understanding that posits just patients who have, and patients who lack, capacity. Given the demands of MCA section 4(6), we can distinguish patients who lack, but once had, relevant capacity, and patients whose values cannot be ascertained. Section III goes on to consider patient values in relation to the concept of the medical decision, which requires to be understood by reference to the multiple ‘stakeholders’ involved in it: the patient herself, the person making the clinical determination, and the authority responsible for resource allocation. Section IV then considers judicial interpretations of how best interests should be understood and applied. It challenges the application of best interests as conceived in *Airedale NHS Trust v Bland*,^11^ suggesting that it is wrong to rely on that case (as Court of Protection judges continue to do) when giving conceptual form to the contemporary best interests standard. It then responds to concerns that best interests and substituted judgment might wrongfully be elided. And finally, it explains the coherence of ‘objective best interests’ being informed or determined by reference to subjective values. The article concludes that it is erroneous to suppose that basing a decision on a patient’s best interests is necessarily something other than deciding in line with what would have been done if she had capacity, and suggests that we should welcome this.

## II. CONCEPTUAL CLARITY ONE: MENTAL CAPACITY LAW’S THREE ADULT PATIENTS

An important legal literature examines the concept of the person.^12^ The implications of being deemed a legal person are profound: persons enjoy legal rights and protections, and suffer obligations and liabilities, that do not apply to mere things. Legal scholarship on personhood examines and incorporates complex philosophical questions, which stand centre stage in a great deal of medico-legal and bioethical debate.^13^ At law it makes a difference to particular rights, freedoms, and liabilities if a person is, for example, an employee, an occupier, a registered medical practitioner, and so on. In each case, we have a concept whose legal significance is of huge import. In the ideal, we want to be able to justify the creation of the concept, and understand its function in practice. We want, say, to establish that it is a good idea for the law to recognise a distinct category called ‘registered medical practitioner’, and to know how her legally defined position distinguishes itself.

This section of the article conceptually examines ‘adult patients’ in mental capacity law. The law provides—on its face—two concepts: those who have, and those who lack, capacity.^14^ Section 1(1) MCA stipulates that we should assume that adults have capacity, and the tests in sections 2 and 3 provide the means by which that assumption might be displaced. Capacity, as is widely observed, is decision-specific, and invites examination of a patient’s practical reasoning. As such, at any given time (depending on the circumstances under issue), English law tells us that we may have one of two patients: either a patient with, or a patient without, capacity.

While this conceptual framing of the patient is not superficial, an examination of the MCA’s demands, and an analysis of the norms and principles that developed both pre and post its coming into force, provide a subtler conceptual landscape.^15^ This is particularly so given the weight afforded to the patient’s own values, most notably—but not uniquely—in section 4(6). Without a more refined comprehension of the concept of the patient, judges risk both *overstating* the importance of autonomy for patients who have capacity, and *underestimating* the weight that should be given to personal values of patients who lack capacity. Mental capacity law, I suggest, presents *three* sorts of patients. First, there are patients who have capacity. Then, within the band of those who lack capacity, there are two further concepts of the patient: those who once had relevant capacity but are deemed now to lack it; and those whose values cannot be (satisfactorily) ascertained. I will consider each category of patient in turn with reference to some of medical law’s most discussed patients.

### A. Patients with Capacity

The patient who has capacity is exemplified in the case of Ms B.^16^ Here, life-preserving interventions were found to be unlawful because the patient refused consent to the invasion of her bodily integrity. The court accepted the health-care team’s good faith in its estimation of Ms B’s best interests (her carers believed that she did not appreciate that she would come to value her life differently after time, and would eventually consider continued existence to be worthwhile). However, even where medical law gives special value to continued life, medico-legal norms have developed to protect a system of value pluralism, where it is recognised that perspectives on a person’s moral, social, spiritual, and other interests legitimately vary. Respect should be given to the specific patient’s conception of her interests including when her life is at stake. In Ms B’s case, the court held that she had capacity, and, therefore, that her view of her interests, and consequent refusal of consent, should prevail over her carers’ view that she failed properly to understand her interests.

The strength of the rights of the patient with capacity is great: canonically, she has an ‘absolute’ right to refuse treatment, or choose between treatments that are offered, for rational or irrational reasons, or for no reason at all.^17^ But part of the misunderstanding that this article seeks to correct obtains in judges and scholars imagining that the patient with capacity is more greatly empowered than the law permits. As such, it is crucial to note two qualifications that apply to the ‘autonomy rights’ of patients with capacity.

First, we have the *Burke* qualification.^18^ In *Burke*, as in Ms B’s case, there was (potentially) a clash between the patient’s and the health-care team’s assessment of what would serve the patient’s interests. Although we can say ‘Mr Burke had capacity’, what we really mean is that he could formulate a reasoned view about his interests, and express a settled position on the values that should direct decisions concerning his care. However, this ‘capacity’ would not of itself equate with determinative decision-making power.^19^ As explained in the next section, his values were not the only ones of relevance. In Mr Burke’s case—unlike Ms B’s—the patient’s values alone could not be determinative because the case concerned a claim *for positive intervention*, rather than non-intervention. As such, the *Burke* qualification reflects limits to the apparent patient with capacity’s rights not because of a finding of ‘mental incapacity’, but because the basis of medical interventions is not founded on the demands of the patient alone. In Ms B’s case, the patient’s perspective could prevail as of absolute right because she demanded non-intervention; there is no correlative absolute right to demand intervention.

The second qualification in regard to ‘patients with capacity’ is found in real-world treatment of patients’ decision-making: ie how things happen in practice, rather than how we might imagine they work if we only refer to the letter of the law.^20^ As medico-legal scholars are informed by bioethical analyses, it is natural that we speak to the concept of the ‘autonomous patient’. While at some level this makes sense, not least as judges also speak to autonomy as a legally protected value, there is not a settled legal definition of autonomy. Rather, through mental capacity law, we can recognise distinct ‘autonomy type’ concerns coming into play.^21^ The paradigm case is not defined by the privileging of a patient’s *choice* or *act of choosing*; rather, laws serve to vindicate her ordering of values and ensuring their application in a determination of her interests.^22^ In philosophical terms, medical law is constructed to give effect to the course of action that best accords with the patient’s second-order preferences; to act in accordance with the values that she, on reflection, would endorse as those that are true for her.^23^

Both the MCA, and a strong body of case law, support an argument that this concept of autonomy—a ‘best desire autonomy’ that functions to support a system of respectful value pluralism—is the gold standard. However, at times, the courts seem simply to endorse a patient’s decision as baldly expressed given her current desires, even if this apparently contradicts what she would, on reflection, choose.^24^ We might reasonably speculate, for example, that a Jehovah’s Witness would find it easier to receive respect for a consent to a life-preserving blood transfusion than for a refusal of such consent, notwithstanding that she would not reflectively endorse the latter decision. Furthermore, the law reports demonstrate that some systems of reasoning are themselves determinative of incapacity, and a patient is held instead to some ideal standard of decision-making; quintessentially, for example, in the case of patients with anorexia nervosa whose second-order desires apparently endorse what would be fatal refusals of nutrition.^25^ Here, the courts impose a rationality that is external to the patient: her capacity is, essentially, denied on the basis that the (apparent) irrationality itself indicates a lack of autonomy, and thus incapacity. In either of these scenarios, while judges might suggest that the ‘absolute’ right to act irrationally distinguishes the patient with capacity from the patient who lacks it, in practice constraints are placed on the rationalities according to which a decision might be made. In blunt terms, patients are ostensibly free to act irrationally, but in reality only in accordance with an unspecified range of ‘irrationalities’. This necessarily tends towards some level of imposition of ensuring that decisions will be made in accordance with some externally identified reasonableness standard: in this sense—if erroneously—there is not an unbounded right of choice even in regard to refusals of treatment in English medical law.^26^

### B. Patients Who Once Had Relevant Capacity, but Now Lack It

The previous discussion suggests that in the paradigm case of medical decision-making, the patient’s reflectively held values are applied to a particular medico-legal question; having capacity is what means that this may happen. This indicates that consent law reflects not unthinking reverence for choice, but respect for the validity of differing value systems. Such concerns can be applied as we move to consider patients who *once* had relevant capacity, but who now lack it. As mental capacity is decision-specific, in a narrow sense (absent a sound advance decision to refuse treatment^27^) there can be no such thing as an enduringly relevant ‘previous capacity’: a necessary condition of a ‘capacitous’ decision is that it *is being* applied contemporaneously. However, as capacity law has been drafted to ensure that we apply where possible the patients’ own values, we can, where patients lack capacity, decide in accordance with reasonable inferences on what the patient would consider her best interests to be. This is not least because of the emphasis given in section 4(6) MCA to finding and acting in accordance with the patient’s values.

To act in accordance with the reflectively endorsed values of a patient who once had relevant capacity but now lacks it, we need to establish how these would apply to the specific decision. A case that highlights how this would work in practice is *Ahsan*^28^ (this case also debunks erroneous judicial pronouncements that best interests decisions for patients in vegetative state (VS) will *always* lead to a ruling in favour of a life-ending removal of treatment, and thus startlingly will *never* be apt for a balance sheet weighing of benefits and burdens).^29^ Mrs Ahsan was in a VS. Although her case was decided before the MCA came into force, Judge Hegarty QC, who heard the case, considered the effect of the Act in his reasoning. Mrs Ahsan was a Muslim, whose family argued should be treated in accordance with Islamic values; meaning specifically that while she would be unaware of it, she should be cared for at home, with her family. The judge agreed, holding that her best interests, given her values when she had capacity to formulate them, impacted on how she should be treated even in a situation where the benefit was both intangible and one of which she would never be cognisant. That she had not expressed a view on the specific question was not relevant: knowledge of her values was sufficient to establish how the particular decision should be made.

### C. Patients Whose Values Cannot Be Ascertained

We may contrast the *Ahsan*-type patient with the patient who lacks capacity and whose values cannot (satisfactorily) be ascertained. There are three distinct situations of relevance here. The first, which conceptually and legally is straightforward, relates to patients whose values cannot be established for compelling practical reasons (eg a patient in an emergency situation, about whom personally little or nothing is known). Secondly, there are patients who as a matter of fact never held (relevant) reflectively endorsed values that can be applied in the circumstances. And thirdly, there are patients who lack capacity, and whose current values are in conflict with their previously held values: section 4(6) MCA requires decision-makers to consider ‘the person’s past *and* present wishes and feelings’, but offers no indication of what to do when these are inconsistent. While the first situation here does not require elaboration, I will discuss the second and third in turn.

Patients who never held (relevant) values can be exemplified through analysis of the case of *Re Y*.^30^ This concerned a woman who lived in an institution, and possessed extremely limited awareness and understanding. Connell J found a means of holding that Ms Y’s best interests were served by her submitting to the painful process of being a bone marrow donor, so that she might help save the life of her sister. He did so in part by *imputing* specific values to the patient, holding that because Ms Y’s *family* could be said collectively to espouse the sort of values that would support a decision to donate, Ms Y herself could be said to do so. Despite such judicial reasoning, in *Y*, we find a patient who cannot in any concrete sense be said to have previously (or currently) held values that can be drawn on and applied in reaching a decision that accords, as best possible, with what *she* would have reflectively endorsed as serving her interests. As such, the application of section 4(6) to such a patient now would either be impossible, or anyway a more complex question than in the more straightforward situation exemplified by *Ahsan*.

Moving to patients whose past and present values conflict, we are presented with still thornier conceptual, legal, and ethical problems. As indicated, the MCA gives little help in adjudicating between the patient’s own inconsistent views. Legal rules regarding anticipatory decision-making suggest that a patient’s more recent values should be prioritised if there is a conflict or apparent change over time.^31^ Equally, in cases of uncertainty, the dominant rule at law is that decision-making should err on the side of life. Consider the sort of patient represented by Ronald Dworkin’s fictional Margo, who suffered dementia and as a result lived a contented life, but not one that her ‘former self’ would have recognised as part of her ‘biography’.^32^ While in Dworkin’s argument Margo’s previous values should be determinative, and lead to a life-ending decision, at law the weight of argument suggests that her current values should be prioritised. In the class of patient exemplified in this article by *Ahsan*, there is a robust rationale for applying previously held, patient-specific values, even while metaphysicists might raise questions about the link between the ‘previous-’ and ‘current person’.^33^ The complexities with a patient who lacks capacity, and whose past and present values conflict, mean that it would not be straightforward to consider her in the same category as *Ahsan*: for current purposes, it cannot be argued that such a patient should be treated simply according to her previous, reflectively endorsed values. However, there is considerable further analysis to be done in relation to that question, beyond the scope of this article.^34^

## III. CONCEPTUAL CLARITY TWO: THE ‘OWNERSHIP’ OF MEDICAL DECISIONS

In linking the previous and the current sections, it bears reemphasising the following: regard for autonomy within capacity law is best conceived as being about respecting a patient’s determination of her interests by reference to her reflectively endorsed values, rather than a more skeletal, non-substantive reverence for bare choice in and of itself. I have alluded to the courts’ concern for patients’ weighting—and then weighing—of reasons, and discussed the subtlety of respecting patients’ values when making findings of incapacity. The law’s paradigmatic position asks that we attempt to apply the patient’s reflectively endorsed values, whether these are inferred directly by asking for consent (in the case of a patient with capacity) or drawn through inferences given facts that can be determined about a patient’s values by other means (in the case of a patient who lacks, but once had, relevant capacity).^35^ In either case, of themselves, the patient’s values should not themselves be displaced at law.

Insofar as the courts have provided any sort of qualification to this (and the fact that they have done so reinforces the view that their concern is with the application of endorsed personal values rather than bare reverence for choice), it may be said to be based on some robust idea of rational scrutiny. For example, a belief that one’s blood is evil,^36^ or that one should not consume calories,^37^ have been found to be inadequate bases on which a patient might assess her interests when deciding whether to give or withhold consent. If theoretically various questions are begged in this approach,^38^ it is at least something that judges acknowledge. Jackson J, for example, states in *A Local Authority v E* that:

I acknowledge that a person with severe anorexia may be in a Catch 22 situation regarding capacity: namely, that by deciding not to eat, she proves that she lacks capacity to decide at all.^39^

In that same case, the judge also applies in his reasoning the perspective on best interests that the patient would, in his view, have espoused had her life been different. He looks for (what he, at least, sees as) the better understood, rationally endorsed patient perspective on her interests: Ms E had started, but failed to complete, a degree in medicine, and Jackson J thought it relevant to note the salience of the value system she might have held had she become a doctor.^40^

Overall, there is good reason to understand mental capacity law as premised on a commitment to understanding patients in a way that incorporates respect for endorsed values; patients’ perspectives on what serves their interests. However, there are some constraints on what values the courts are willing also to endorse. And crucially, it does not follow from the commitment to patient-centredness that a treatment should be given simply because a patient wants it, or it is found that she would want it if she had capacity. We thus need a clear understanding of the law’s framing of the *medical decision*, which will then allow us to question judicial attempts to drive a wedge between law’s treatment of patients who have, and patients who lack, capacity.

### A. ‘Ownership’ of Medical Decisions

There is a detailed legal literature on the ‘anatomy’ of medical decision-making, from Ian Kennedy’s early framing of the field,^41^ through Penney Lewis’s study in the criminal law setting,^42^ to a wider-reaching contemporary scholarship pioneered by scholars including Sara Fovargue and Alex Mullock.^43^ Rather than answer here the general question ‘what is a medical decision?’^44^, my aim is to deconstruct the idea of medical decision-making by reference to specific ‘stakeholders’ and their relative ‘ownership’ of a decision’s different components.

To identify the stakeholders, it is useful to revisit *Burke*. Lord Phillips, in the Court of Appeal, stated that:

The proposition that the patient has a paramount right to refuse treatment is amply demonstrated by the authorities… . The corollary does not, however, follow, at least as a general proposition. Autonomy and the right of self-determination do not entitle the patient to insist on receiving a particular medical treatment regardless of the nature of the treatment. Insofar as a doctor has a legal obligation to provide treatment this cannot be founded simply upon the fact that the patient demands it. The source of the duty lies elsewhere.^45^

These *dicta*, if cryptic, are consistent with settled legal principle. English law, in its default, protects patients from interference: lawful reason is required before it is permissible to breach a person’s bodily integrity. Depending on the circumstance, different legal criteria need to be met before a person’s bodily integrity may be breached. Consent may be one such criterion. But in many circumstances consent alone is insufficient to justify intervention. The sorts of interventions that might occur within health care are subject to the ‘medical exception’: ie they are *prima facie* unlawful acts that are permissible because it is in the public interest to allow them to happen.^46^ As Richard Huxtable shows, there may be a requirement of consent, but this is not adequate to explain the concept of the lawful medical intervention.^47^ To understand the ‘stakeholders’ within a lawful decision to *provide* health care, and explain the interaction and nature of their respective stakes, Huxtable’s analysis leads to the following triad (which does not directly reflect his own presentation of the ideas, but which follows from his analysis).

For treatment to be lawful it is requisite that:

- It is established to reflect, or at least be consistent with, the patient’s personal view of her interests: this may be established through gaining consent, or by reference to proven facts about the patient’s values.
- It is judged by reference to professional opinion to be in the patient’s best interests: this will be established by reference to the doctor(s) agreeing that the intervention is indicated as a worthwhile intervention because of the benefits—whether therapeutic or otherwise—that it will provide.^48^
- It is judged, by reference to principles of sound public decision-making, to be worth funding through the health care system: this will be established by the particular resource allocation model that governs access to treatment.^49^

Judges may not be explicit about this basis for lawful provision of health care (not least as each component may not require a legal determination in a given case). But each of these clusters is a necessary component of lawful medical decision-making. For receipt or provision of treatment, patient-centred care does not equate with care being defined simply by reference to the patient’s personal values. While Ms B’s case exemplifies a purely patient-centred right to refuse treatment, Mr Burke’s case and its surrounding doctrine exposes how more is at play than patient autonomy when we look to positive medical decision-making. Baroness Hale is clear about this in *Aintree*, and thereby of the equivalent situations of patients who have and who lack capacity:

The purpose of the best interests test is to consider matters from the patient’s point of view. That is not to say that his wishes must prevail, any more than those of a fully capable patient must prevail. We cannot always have what we want. Nor will it always be possible to ascertain what an incapable patient’s wishes are. […] But in so far as it is possible to ascertain the patient’s wishes and feelings, his beliefs and values or the things which were important to him, it is those which should be taken into account because they are a component in making the choice which is right for him as an individual human being.^50^

This analysis, placing the patient’s perspective as *a component* of a wider determination of what might be a viable medical decision positions itself in happy consonance with how the General Medical Council frames medical decision-making as a joint enterprise, involving ‘patients and doctors making decisions together’.^51^ Perhaps especially within the context of publicly funded health care, there is recognition of a need to defer to doctors’ expertise; for treatment to be warranted in their expert judgment. This concern, as Huxtable suggests, rests both on the need for patients not to be able to demand that doctors cause them harm,^52^ and on a need to ensure that resources are used as effectively as possible. A patient might understand the properties and effects of morphine, and come to the view that it would serve her best interests to take it. It does not follow that on the basis of her determination her doctor is—or should be—legally obliged to prescribe it!

So, while the metaphor is crude, we can picture the ‘ownership’ of a medical decision by reference to who has a stake in it. If it is non-intervention or a choice between different offered treatments, in principle at least, we can look simply to the patient’s values: this might be through asking for her consent (as eg in the case of Ms B) or by reference to what we know about the patient (eg we would know not to provide a blood transfusion to a patient who lacks capacity if we knew that she was a committed Jehovah’s Witness). If it is to establish which potential treatments might be offered, the doctor as an expert in medicine, and the state as a provider and distributor of limited resources, also hold stakes: here, we look to the need of the patient, as judged ‘externally’, and to what might be available as a viably funded treatment.

### B. The Value Basis of Decisions: Clarity on the Parity Between Patients with and without Capacity

The analysis thus far has explained the ‘shape’ of different patients within mental capacity law, and outlined the ‘ownership’ of medical decisions through reference to the stakeholders in them. We can conclude this section by advancing some overall foundational premises for the critical argument advanced below.

The concept of the patient with capacity—the ‘autonomous’ or ‘competent patient’—is sometimes one whose standing is overstated.^53^ ‘Absolute’ rights of non-interference do not translate into absolute rights to claim, either against doctors in the face of contrary (and reasonable) professional judgment, or against the state in the face of lawful resource allocation decisions. The better understanding of the function of receiving consent, especially in a system that looks to patient values and ensured patient understanding,^54^ is that it allows the patient to explain from a personal perspective what does or does not serve her interests. Finally, we have seen that the ‘absolute’ deference offered to patient’s reasons, be they rational or irrational, is not always reflected in case law: patients may not obviously fail the MCA tests for capacity, and yet may still be found to lack decision-making capacity.

The various conceptual subtleties presented above allow us to see a parity that is not always obvious between patients who have capacity and those who now lack, but once had, relevant capacity. With *Aintree*, we have authority at the highest level that patients’ applicable values survive their loss of capacity and require to be respected over and above any claims about ‘objective medical interests’. Baroness Hale rejects the legal construction of a ‘reasonable patient’ whose interests apply in any case of incapacity^55^; a point to which we return in Section IV. With section 4(6) MCA, and the common law developments regarding patients who lack capacity, there is a great emphasis on the need to weigh factors in accordance with patients’ pre-existing values. The overall situation suggests that within the legal framing of the medical decision, the patient-centred component is aimed at safeguarding—and to the extent that is compatible with professional judgment and resource allocation concerns, vindicating—the patient’s reflectively endorsed perspective. Patient-centred decision-making protects patients’ values whether they have or lack capacity, and it does so in a framing that in part accepts that patients’ interests are defined specifically by reference to their own values.

The outlier in law’s framing of patients, therefore, is not ‘the incompetent patient’ as contrasted with the ‘competent patient’. Rather, the difficulties come with patients whose values cannot be ascertained, be that because of an emergency situation, because of the patient never having had or demonstrated applicable values, or because of an internal conflict of personal values. But for patients who once had, and now lack, relevant capacity, this problem does not arise. As such, it might be argued that the onus is on the decision-maker to establish that the patient’s values should be set aside if that is her view. However, the Court of Protection has developed a problematic situation wherein the rights of people who lack capacity have been weakened. The following section responds to this.

## IV. TOWARDS LEGAL CLARITY ON MEDICAL DECISION-MAKING

My argument now responds to developing Court of Protection ‘wisdoms’, highlighting three related areas of judicial reasoning. I first question the ongoing application of *Bland*^56^ in contemporary judicial reasoning. I then consider concerns that decision-making for patients who lack capacity risks wrongfully becoming a substituted judgment test. Finally, I explore the apparent apprehension that if subjectivity seeps into decision-making for patients who lack capacity, this undermines demands to look to ‘objective best interests’.

### A. *Best Interests: No Longer a* Bland *Idea*

A defining feature of medical jurisprudence is the extent to which it is a product of evolving judge-made law.^57^ Historical landmark cases in health care law are often bare on legal authority, draw explicitly from non-legal norms (eg ones derived from medical understandings or public reports), and explicitly tweak and enhance the dominant rules, standards, and principles as time goes on. For example, consider how Thorpe J provided the test for incapacity in *Re C*. The legal authority comes from just *Bland* and *Re F*^58^ (neither of which concerns a patient whose incapacity was in doubt), *Re T*, and the judge draws explicitly from a model of capacity advanced by a medical professional. Or consider the development of negligence-based protections of informed consent, culminating in the botch-job *ratio* of *Chester v Afshar*^59^ and the more straightforward, if distinctly radical, decision in *Montgomery*.^60^

This characteristic of medical law as a judge-led field is remarkable in part because—in Kenneth Veitch’s framing^61^—it represents a claiming of ‘jurisdiction’ by the courts to determine matters of professional and ethical importance. The courts have assumed authority and developed rules and principles of governance in response to advancing medical possibility and evolving social and ethical norms. But it is more remarkable still because while medical law may be defined by reference to the central role of judges, it also repeatedly offers challenges to standard understandings of constraints on judicial decision-making. This applies in relation both to statutory interpretation and the application of precedent authority.

For example, *Quintavalle^62^* is literally a textbook illustration of ‘purposive’ judicial reasoning, given to first year law students to demonstrate judicial departure from literal readings of statutes.^63^ And regarding common law developments, we see that higher court decisions come to stand as formally good, but practically bad, law. Strikingly, the law governing best interests is pre-eminent in this regard. Best interests began as a very ‘*Bolam*-esque’ idea; in *Re F* and *Bland* it is doubly medicalised, in essence eliding ‘best interests’ and ‘best medical interests’, and offering a standard whose substantive content is largely left to the judgment of medical experts. Through developments in the late 1990s and 2000s, however, judges pushed away from both features of medicalisation. In their place, a patient-focused standard emerged comprising the ‘Bolam’ aspect (ie, in practitioners’ expert judgment, is the treatment clinically indicated?) but also a fuller concern for interests beyond those that are medical. And with this development, the courts produced a reframing that denied the place of doctors as the final or necessarily best judges of best interests.^64^

*Bland* of course remained ‘good law’ even while the flesh it had put on the bones of best interests was stripped away and a substantively distinct body of legal rules was created. So formally, to the extent that it is consistent with by the MCA, *Bland* still stands. Yet the development of the substantive idea of best interests clearly shows, over time, a radical reformulation of the standard explicated in *Bland*. While the *rationes* of that case, narrowly conceived, might endure, much of the detail and nuance of emphasis simply do not. The judicially created, patient-centred best interests standard that emerged over time, focusing on the values of the patient, and her global rather than just her medical interests, is distinct from the judicial framing of best interests in *Bland*, and rests on alternatively conceived foundations.

I therefore suggest that when judges, health care practitioners, and other decision-makers wish to substantiate an understanding of best interests, or apply the standard, it is wrong for them to have recourse to the detail of *dicta* in *Bland*. Controversial as such a claim must appear, my argument is first that *Bland*’s narrow understanding of best interests at common law became redundant through legal evolution. And perhaps more compellingly, the MCA necessarily renders the earlier standard inapplicable, and replaces it with a fuller, richer, less bland, alternative. Even if lower-courts’ judicial law-making does not strictly undermine the force of *Bland*, the MCA necessarily does so. *Bland* is perhaps medical law’s landmark case, but its substantive explication of principle is, in a very real legal sense, outdated. When the Court of Protection approaches questions of best interests, it must do so with the more patient-centred idea, rather than purport to have serious recourse to understandings that have long been set aside both by judges and by Parliament.

### B. When Words Collide: Overcoming Concerns about ‘Substituted Judgment’

To highlight my concerns about the problematic application of *Bland*, I will focus on Baker J’s reasoning in *M* on how best interests must be distinguished from a substituted judgment standard. His decision builds directly on Lewison J’s in *P*, advancing the position, essentially, that it cannot make sense to allow the parity that I have described above between patients who have capacity, and those who once had relevant capacity. Baker J holds:

Lord Goff stated [in *Bland*] at p 871, that the so-called ‘substituted judgment’ test adopted in most American courts—whereby ‘the court seeks, in a case in which the patient is incapacitated from expressing any view on the question whether life-prolonging treatment should be withheld in the relevant circumstances, to determine what decision the patient himself would have made had he been able to do so’—did not form part of English law in relation to incompetent adults ‘on whose behalf nobody has power to give consent to medical treatment’.^65^

He continues later in the judgment:

It is important to note that, while any decision-maker, including a judge, is under an obligation to consider P’s [the patient’s] wishes and feelings, and the beliefs, values and other factors he would have taken into account if he had capacity, the decision must be based on P’s best interests and not on what P would have decided if he had capacity.^66^

Baker J argues that this view is supported by the reasoning of Lewison J in *P*, the explanatory notes of the original Mental Capacity Bill, and Lord Goff’s *dicta* in *Bland*. He also refers to the MCA Code of Practice, which ‘confirmed’, he reasons that we cannot in a best interests evaluation focus on what the patient would have decided. The crucial lines within the passage that he quotes from the Code are:

[The patient’s] wishes and feelings, beliefs and values will not necessarily be the deciding factor in working out their best interests. [… T]he final decision must be based entirely on what is in the person’s best interests.^67^

Baker J’s reasoning has superficial appeal, but is flawed. First, as I have indicated, strong reliance on substantive claims advanced in *Bland* are problematic: Lord Goff explicated principle in a way that is not even reflected in subsequent common law developments, and whose effect is negated by the MCA. For the reasons presented in the previous section, we should doubt the reliance that may be given to substantive components of many *dicta* in *Bland*. Demedicalising developments at common law, and then in the MCA, provide compelling reason not to have determinative recourse to an explication of the best interests standard that no longer holds. Secondly, and equally, at law the notes of the original Mental Capacity Bill are not determinative. A judge might refer to them for whatever persuasive force they may have, and we see below that Lady Hale draws from them too in her opinion in *Aintree*.^68^ But they are in no hard way a *constraint* on best interests determinations, and where they conflict with a proper reading of the law as subsequently enacted, their force is to be questioned. Thirdly, and perhaps the most important point here, which rests on the analysis in Sections II and III of this article, is that Baker J apparently misunderstands the passage that he takes from the Code of Practice, which aligns itself with a distinct understanding to that attributed to Lord Goff’s. Lord Goff’s point, as cited by Baker J, is that English law provided no means of introducing a substituted judgment. The Code of Practice, by contrast, is explaining a separate point: that a best interests decision is informed by, but not only by, the patient’s values.

To explain this third point more fully, recall how the above analysis shows that a patient’s views on her interests will not necessarily be determinative of what treatment is ultimately administered. Yet that analysis showed that this is true for patients with capacity too. As we have seen, a positive medical decision is best conceived as comprising a combination of three perspectives on what serves the patient’s best interests: her own, reflectively endorsed view, the professional’s expert view, and the public interest view. So for *any* patient, personal values cannot be said necessarily to be the deciding factor. Baker J’s reasoning represents a misunderstanding of the medical decision, which leads to an illusory distinction between patients who have and patients who lack capacity, and provides an undue licence then to disregard the patient’s own values in order to assert the pre-eminence of some externally preferred value (in the case of *M*, the imposition of a rigid sanctity of life ethic^69^).

Two further points may be noted here. First, scholarly analyses of best interests and substituted judgment standards demonstrate that each of these terms designates a wide range of often overlapping ideas. John Phillips and David Wendler present a useful conceptual analysis of substituted judgment, and a defence of their preferred understanding.^70^ In their argument, we find claims about necessary distinctions between best interests and substituted judgment. However, given my analysis above, I would doubt that what some protagonists label ‘substituted judgment’ is not conceptually identical to what others designate ‘best interests’. Depending on how it is cashed out, the patient-centred decision-making standard described in this article for patients who lack but once had relevant capacity could as comfortably be labelled best interests as substituted judgment. Overall, the substance here is more important than making a distraction of the label.

A key reason, though, that we might prefer the label ‘best interests’ is regard to patients whose values cannot be ascertained. For them, the parity that I have outlined with patients who have capacity does not exist. As explained above, this may be because we do not know anything about the patient personally, because the patient simply never had the capacity to formulate the relevant values, or because she espouses inconsistent values. In any such case, purporting to apply the patient’s endorsed values—to provide a substituted judgment—would be an exercise in fiction.^71^ So without getting too distracted by labels, we can see why best interests could be the more appropriate term, while also accepting that where it is possible, it should operate as a substituted judgment test that applies the reflectively endorsed values that the patient would bring to the decision-making.

This position is consistent with Lady Hale’s analysis in *Aintree*. As she states:

The advantage of a best interests test was that it focused on the patient as an individual, rather than the conduct of the doctor, and took all the circumstances, both medical and non-medical, into account… . But best interests should also contain ‘a strong element of “substituted judgment” ’ … taking into account both the past and present wishes of the patient as an individual, and also the factors which he would consider if able to do so … . This might include ‘altruistic sentiments and concern for others’… . This is, as the Explanatory Notes to the Bill made clear, still a “best interests” rather than a “substituted judgment” test, but one which accepts that the preferences of the person concerned are an important component in deciding where his best interests lie.^72^

And as she goes on to hold:

[I]n considering the best interests of this particular patient at this particular time, decision-makers must look at his welfare in the widest sense, not just medical but social and psychological … they must try and put themselves in the place of the individual patient and ask what his attitude to the treatment is or would be likely to be.^73^

These *dicta* support and are supported by the analysis that I have provided throughout this article. There is, however, one key point of at least apparent discord between my analysis and Lady Hale’s presentation of the framing that mental capacity law gives to patients, respectively, with and without capacity. This comes out in her discussion of ‘unwise’ decisions. Lady Hale holds that:

A person who has the capacity to decide for himself can of course make decisions which are not in his own best interests and no doubt frequently does so. Indeed, the Act provides that a person is not to be treated as unable to make a decision simply because he makes an unwise one: section 1(4). But both at common law and under the Act, those who act or make decisions on behalf of a person who lacks capacity must do so in his best interests: section 1(5).^74^

While there is intuitive appeal to the reasoning here, it becomes apparent that the ideas that are expressed are not straightforward. First, Lady Hale’s suggestion that a person with capacity might act against her best interests relates to an alternative concept of best interests: a patient with capacity may act in a way that is in some senses harmful, but the crucial point is that insofar as they are applicable, the decision is respected because an *overall* understanding of her interests is informed by reference to the application of her values to the decision. This is no different for patients who lack, but once had, relevant capacity. So a Jehovah’s Witness, whether he has or lacks capacity, may see effected the ‘unwise’ decision, which many would say is ‘not in his own best interests’, not to receive a blood transfusion. But within law’s framing, as explained above, this is precisely about understanding the patient’s values as being incorporated within an assessment of his interests, regardless of whether he has or lacks capacity. In other words, the way best interests has come to be constructed at law does, in effect, mean that patients with capacity are being treated in their best interests. It is hard to see, in short, how or why the statutory language suggests that a ‘wise’ decision and a ‘best interests’ decision are somehow synonymous: an ‘unwise’ decision may well be precisely what serves a patient’s best interests in the sense that legal principle would have us understand that standard.

As such, and consistently with Lady Hale’s wider analysis in *Aintree*, I have argued that there should be parity in mental capacity law’s treatment of patients’ values, if these can be established, regardless of whether they currently have or lack capacity. A consequence of this, and the overall dissection of the concept of the medical decision, is that it is proper to understand decision-making for patients who have capacity as being about an assessment of their overall best interests, given the combination of their personal values, the clinical judgment, and the public interest. As a matter of law, patient-centred care requires a determination on best interests that incorporates the values of the patient who lacks capacity, where possible, as fully as would be the case for a patient who has capacity: the only difference is the route taken to establishing what those values are.

### C. Reconciling Subjectivity and ‘Objective Best Interests’

Against what has been argued, a further means of problematising the more patient-centred approach to best interests lies in arguments that best interests is an objective—not a subjective—standard. As such, the apparent logic suggests, it would be erroneous to focus on the values of the subject (ie the patient) and we should instead look to objective (ie ‘true’) values.^75^

We might begin by noting that in *Aintree* Lady Hale holds that the standard should indeed be patient-focused, stating: ‘[With the idea] that the test of the patient’s wishes and feelings was an objective one, what the reasonable patient would think, again I respectfully disagree.’^76^ In advancing a justification of this position, I would endorse Lady Hale’s reasoning, which is spelled out above.^77^ And I would supplement Lady Hale’s argument with a further point about the role of ‘objectivity’. The demands to find what is objectively in the patient’s best interests should not be equated with a demand to find some monistic, universal value and apply it to the individual just because she lacks capacity. Indeed, the idea that such a value exists within medical law stands at odds with its clear and settled value-agnosticism.^78^ The objectivity that the courts should be aiming at obtains in making a finding of fact about what the patient’s relevant, endorsed, subjective values are.^79^

Assume, for example, that a court establishes as a matter of fact that a patient was a committed Jehovah’s Witness. It follows from this that, objectively, it is not in her best interests to receive blood. This is not because it is objectively true that people should not ‘consume’ blood. Rather, the court makes its finding on best interests on the basis of an objectively verifiable understanding of the subjective values of the patient: it is an objective fact that the patient’s values are incompatible with receiving the transfusion. This is consistent with the patient-centred approach advanced by Lady Hale, but makes clearer why it is wrong for judges to impose external values (such as a sanctity of life ethic) on the simplistic basis that such positions are ‘objective’.

## V. CONCLUSIONS: ACHIEVING PATIENT-CENTRED PRACTICE WITHIN EXISTING MENTAL CAPACITY LAW

The above analysis has been advanced in a conceptual and theoretical voice, but draws ultimately from standards, rules, and principles found at law. To argue that the law provides an ethically desirable standard for decision-making would require its own sustained analysis. It is worth noting, however, that all of the above can be seen to rest upon a principled commitment to respecting and accommodating value pluralism: a cardinal ethico-legal feature within the evolving canons of medical law. More compellingly, what this article shows is that, without revision, English mental capacity law demands that decision-makers take patient-centred approaches in all cases, giving parity to the determinative strength of a patient’s own values, if these can be established, whether she has, or lacks, capacity. The target of my critique is erroneous interpretation and application of the law, rather than the law itself. My argument, founded on an explanation of the ‘shape’ that law gives to patients and medical decision-making, provides a legally and conceptually robust understanding and framework for mental capacity law cases. The core concern in patient-centred health care and law is the vindication of patients’ endorsed values; not a fetishisation of choice, or a concomitant neglect of personal values where choice is not possible. As we have seen, patients’ values, whether they have or lack capacity, are not all that is relevant. But patients’ values, alongside professional and public judgment, must be taken seriously.

## Footnotes

1 *Re P (Statutory Will)* [2009] EWHC 163 (Ch); [2010] 2 WLR 253.

2 ibid, paras 36–45.

3 *Re M (Adult Patient) (Minimally Conscious State: Withdrawal of Treatment)* [2011] EWHC 2443 (Fam); [2012] 1 WLR 1653.

4 See, respectively, *Re P* (n 1), para 36 and *Re M*, ibid, para 85. My own view, explored below, is that the doctrine, if not the practice, suggests an evolution of which the MCA forms a part. In regard to patient participation, including in the application of the patient’s beliefs and values, see also the analysis in Mary Donnelly, ‘Best Interests, Patient Participation and the Mental Capacity Act 2005’ (2009) 17(1) Med LR 1.

5 Throughout this article, I use ‘values’ to encompass also wishes, feelings, and beliefs: this is for brevity of expression, and should not be taken to imply a view that these four different concepts are reducible to one.

6 See especially Hayden J’s judgment in another MCS case, *M v Mrs N and Others* [2015] EWCOP 76, where a determination was made not to continue provision of treatment because, as held at para 75, ‘it would be disrespectful to Mrs. N to preserve her further in a manner I think **she** would regard as grotesque’ (emphasis in original).

7 *R (On the Application of Oliver Leslie Burke) v The General Medical Council* [2005] EWCA Civ 1003.

8 *Aintree University Hospitals NHS Foundation Trust v James* [2013] UKSC 67.

9 John Coggon, ‘Assisted Dying and the Context of Debate: ‘Medical Law’ *versus* ‘End-of-Life Law’ ’ (2010) 18(4) Med LR 541; Richard Huxtable, ‘Autonomy, Best Interests and the Public Interest: Treatment, Non-treatment and the Values of Medical Law’ (2014) 22(4) Med LR 459; Jonathan Herring and Jesse Wall, ‘Autonomy, Capacity and Vulnerable Adults: Filling the Gaps in the Mental Capacity Act’ (2015) 35(4) LS 698.

10 See especially the Select Committee on the Mental Capacity Act 2005, *Mental Capacity Act 2005: post-legislative scrutiny* (HL 2013-14, 139); United Nations Convention on the Rights of Persons with Disabilities.

11 *Airedale NHS Trust v Bland* [1993] AC 789.

12 For a clear overview and critical analysis, see Ngaire Naffine, *Law’s Meaning of Life* (Hart 2009).

13 This is true especially in regard to beginning and end of life questions: see eg the debates between John Finnis and John Harris in chs 1–6 of John Keown (ed), *Euthanasia Examined* (CUP 1997); Sheelagh McGuinness and Marie Fox, ‘The Politics of Muddling Through: Categorising Embryos’ in Catherine Stanton and others (eds), *Pioneering Healthcare Law: Essays in Honour of the Work of Margaret Brazier* (Routledge 2016).

14 The conceptual claim here is not unsettled by the judicial activism that has led to the emergence of the category of vulnerable adult, who has mental capacity but is nevertheless denied decision-making capacity (see eg *Re SA (Vulnerable Adult with Capacity: Marriage)* [2006] 1 FLR 867). There are, however, interesting upshots to the Courts’ development of the inherent jurisdiction that warrant analysis that cannot be accommodated in the current article, but see Michael Dunn, Isabel Clare and Anthony Holland, ‘To Empower or to Protect? Constructing the ‘Vulnerable Adult’ in English Law and Public Policy’ (2008) 28(2) LS 234; Hazel Biggs and Caroline Jones, ‘Legally Vulnerable: What Is Vulnerability and Who Is Vulnerable?’ in Michael Freeman, Sarah Hawkes and Belinda Bennett (eds), *Law and Global Health* (OUP 2014).

15 See also Huxtable (n 9).

16 *Ms B v An NHS Hospital Trust* [2002] EWHC 429 (Fam); [2002] 2 All ER 449.

17 *Re T (Adult: Refusal of Treatment)* [1993] Fam 95; MCA s 1(4).

18 *Burke* (n 7).

19 Philip Bielby, ‘The Conflation of Competence and Capacity in English Medical Law: A Philosophical Critique’ (2005) 8(3) Med Health Care Philos 357.

20 For a full analysis to substantiate the claims made here, see John Coggon, ‘Varied and Principled Understandings of Autonomy in English Law: Justifiable Inconsistency or Blinkered Moralism?’ (2007) 15(3) Health Care Anal 235.

21 ibid. See also John Coggon, ‘Anorexia Nervosa, Best Interests, and the Patient’s Human Right to a ‘Wholesale Overwhelming of her Autonomy’: *A Local Authority v E* [2012] EWHC (CoP) [2012] HRLR 29’ (2014) 22(1) Med LR 119; John Coggon, ‘Alcohol Dependence and Anorexia Nervosa: Individual Autonomy and the Jurisdiction of the Court of Protection: *An NHS Foundation Trust v Ms X* [2014] EWCOP 35; (2014) 140 BMLR 41’ (2015) 23(4) Med LR 659.

22 Coggon (n 20); Huxtable (n 9); Herring and Wall (n 9).

23 Harry Frankfurt, ‘Freedom of the Will and the Concept of a Person’ (1971) 68(1) J Phil 5; Gerald Dworkin, *The Theory and Practice of Autonomy* (CUP 1988).

24 For an interesting example, see *Re W (Adult: Refusal of Medical Treatment)* [2002] EWHC 901 (Fam); [2002] MHLR 411.

25 See eg *A Local Authority v E* [2012] EWHC 1639 (CoP); [2012] HRLR 29; Tony Hope and others, ‘Agency, Ambivalence and Authenticity: The Many Ways in which Anorexia Nervosa Can Affect Autonomy’ (2013) 9(3) Int JLC 20.

26 For a fuller substantiation and analysis, see Coggon (n 20).

27 As defined in MCA ss 24–26.

28 *Ahsan v University Hospitals Leicester NHS Trust* [2006] EWHC 2624 (QB); [2007] PIQR P19.

29 At para 35 of *Re M* (n 3), Baker J states that: ‘In vegetative state cases, the balance falls in one direction in every case—in favour of withdrawal.’ The apparent confusion here likely results from the court’s conflating a universal comment on patients’ best interests with an observation born of the general truth that when health care teams, with family support, apply to the courts for an application on the lawfulness of withdrawal of life-sustaining treatment, that this will be granted.

30 *Re Y (Mental Patient: Bone Marrow Donation)* [1997] Fam 110.

31 *HE v A Hospital NHS Trust* [2003] EWHC 1017 (Fam); [2003] 2 FLR 408; MCA s 25(2)(c).

32 Ronald Dworkin, *Life’s Dominion* (Alfred Knopf 1993) 218–37.

33 Cf Jeff McMahan, *The Ethics of Killing–Problems at the Margins of Life* (OUP 2002).

34 I am grateful to Neil Allen for pushing the importance of this point and highlighting the need for further attention to be given to the important questions raised by the law here.

35 See also the analysis in John Coggon, *What Makes Health Public?* (CUP 2012), ch 10.

36 *X NHS Trust v T (Adult Patient: Refusal of Medical Treatment)* [2004] EWHC 1279 (Fam); [2005] 1 All ER 387.

37 *A Local Authority v E* (n 25).

38 Coggon, ‘Anorexia Nervosa, Best Interests, and the Patient’s Human Right to a ‘Wholesale Overwhelming of her Autonomy’ ’ (n 21).

39 *A Local Authority v E* (n 25), para 53.

40 ibid, paras 78–79.

41 Ian Kennedy, ‘What is a Medical Decision?’ in Ian Kennedy, *Treat Me Right: Ethics in Medical Law and Ethics* (Clarendon Press 1988).

42 Penney Lewis, ‘The Medical Exception’ (2012) 65 CLP 355.

43 Sara Fovargue and Alexendra Mullock (eds), *The Legitimacy of Medical Treatment: What Role for the Medical Exception* (Routledge 2015).

44 This question is examined more fully in John Coggon, ‘Comments and Reflections on “Proper Medical Treatment”: A Case for Coherent Inconsistency’ in ibid.

45 *Burke* (n 7), para 31.

46 *R v Brown* [1994] 1 AC 212 (HL).

47 Huxtable (n 9).

48 *Aintree* (n 8); paras 37–41 (Lady Hale), where it is made clear that a broad understanding of welfare requires that doctors include in an evaluation of treatment its non-therapeutic benefits in an assessment of its efficacy and worth.

49 *Montgomery v Lanarkshire Health Board* [2015] UKSC 11; [2015] 2 WLR 768, para 75.

50 *Aintree* (n 8), para 45.

51 General Medical Council, *Consent: Patients and Doctors Making Decisions Together* (GMC 2008).

52 Consider this requirement too in the light of discussions such as José Miola’s analysis of conscience in health care: José Miola, ‘Making Decisions about Decision-Making: Conscience, Regulation, and the Law’ (2015) 23(2) Med LR 263.

53 Cf Charles Foster, *Choosing Life, Choosing Death: The Tyranny of Autonomy in Medical Ethics and Law* (Hart 2009).

54 *Montgomery* (n 49).

55 *Aintree* (n 8), para 45.

56 *Bland* (n 11).

57 Kenneth Veitch, *The Jurisdiction of Medical Law* (Ashgate 2007).

58 *Re F (Mental Patient: Sterilisation)* [1990] 2 AC 1.

59 *Chester v Afshar* [2004] 4 All ER 587.

60 *Montgomery* (n 49).

61 Veitch (n 57).

62 *R (Quintavalle) v Secretary of State for Health* (2003) 2 All ER 113.

63 See eg Gary Slapper and David Kelly, *The English Legal System—16^th^ edn* (Routledge 2015) 98–101.

64 See especially *Re Y* (n 30); *Re A (Medical Treatment: Male Sterilisation)* [2000] 1 FCR 193; *Re S (Adult Patient: Sterilisation)* [2001] Fam 15; *An NHS Trust v A, SA* [2005] EWCA Civ 1145; *Ahsan* (n 28).

65 *Re M* (n 3), para 71.

66 ibid, para 81.

67 The passage from which the judge is quoting is found in Department for Constitutional Affairs, *Mental Capacity Act 2005—Code of Practice* (TSO 2007), para 5.38.

68 See text to n 73.

69 See Richard Huxtable, ‘ ‘In a Twilight World’? Judging the Value of Life for the Minimally Conscious Patient’ (2013) 39(9) JME 565.

70 John Phillips and David Wendler, ‘Clarifying Substituted Judgement: The Endorsed Life Approach’ (2014) 41(9) JME 723.

71 See also Louise Harmon, ‘Falling Off the Vine: Legal Fictions and the Doctrine of Substituted Judgment’ (1990) 100(1) Yale LJ 1.

72 *Aintree* (n 8), para 24.

73 ibid, para 39.

74 ibid, para 23.

75 See eg Lewison J’s reflections in *Re P* (n 1), para 37 and Baker J’s in *Re M* (n 3), para 81.

76 *Aintree* (n 8), para 45.

77 See quoted text to n 50.

78 Coggon (n 9).

79 John Coggon, ‘Best Interests, Public Interest, and the Power of the Medical Profession’ (2008) 16(3) Health Care Anal 219.

